# Brain activity pattern changes after adaptive working memory training in multiple sclerosis

**DOI:** 10.1007/s11682-018-9984-z

**Published:** 2018-10-30

**Authors:** Laura Bonzano, Ludovico Pedullà, Matteo Pardini, Andrea Tacchino, Paola Zaratin, Mario Alberto Battaglia, Giampaolo Brichetto, Marco Bove

**Affiliations:** 1 Department of Neuroscience, Rehabilitation, Ophthalmology, Genetics, Maternal and Child Health, University of Genoa, Genoa, Italy; 2 Magnetic Resonance Research Centre on Nervous System Diseases, University of Genoa, Genoa, Italy; 3 Department of Experimental Medicine, Section of Human Physiology, University of Genoa, Viale Benedetto XV 3, 16132 Genoa, Italy; 4 Italian Multiple Sclerosis Foundation, Scientific Research Area, Genoa, Italy; 5 IRCCS Ospedale Policlinico San Martino, Genoa, Italy; 6 Department of Life Science, University of Siena, Siena, Italy

**Keywords:** Cognitive rehabilitation, fMRI, Multiple sclerosis, Working memory, Adaptive training, Cognitive reserve

## Abstract

Cognitive impairment and related abnormal brain activity are common in people with multiple sclerosis (PwMS). Adaptive training based on working memory (WM) has been shown to ameliorate cognitive symptoms, although the effects at a neural level are unclear. The aim of this study was to expand the existing research on the effects of an adaptive WM rehabilitative intervention on brain functional activity in PwMS. A sample of eighteen PwMS performed an 8-week home-based cognitive rehabilitation treatment based on adaptive WM training. PwMS were assessed before and after treatment using a validated neuropsychological battery and undergoing an fMRI session while carrying out a cognitive task (i.e., Paced Visual Serial Addition Test - PVSAT). fMRI activations were compared to the activation pattern elicited by eighteen matched healthy subjects performing the same task. At baseline, we found abnormal brain activity during PVSAT in PwMS when compared to healthy subjects, with a pattern including several bilateral activation clusters. Following rehabilitation, PwMS improved cognitive performance, as evaluated by the neuropsychological battery, and showed a different activation map with clusters mainly located in the right cerebellum and in the left hemisphere. The only significant cluster in the right hemisphere was located in the inferior parietal lobule, and the BOLD signal extracted in this area significantly correlated with cognitive performance both before and after the treatment. We suggest that WM training can improve the cognitive performance and reduce the abnormal activation of PwMS by partially maintaining or even restoring brain cognitive function.

## Introduction

Cognitive impairment is a common symptom in multiple sclerosis (MS), with more than half of the people with MS (PwMS) showing cognitive deficits at formal neuropsychological testing (Amato et al. 2006a, b), especially impacting information processing speed and attentional abilities (DeLuca et al. 2015).

fMRI studies have explored cognitive processes in PwMS examining different functions, such as working memory, attention, and executive functions (Chiaravalloti and DeLuca 2008). In particular, the Paced Auditory Serial Addition Test (PASAT) (Audoin et al. 2005a, b; Forn et al. 2006; Mainero et al. 2004), the Paced Visual Serial Addition Test (PVSAT) (Bonzano et al. 2009), (i.e., the visual analogue of the PASAT (Nagels et al. 2005)), and the *N*-back task (Amann et al. 2011; Cader et al. 2006; Forn et al. 2007; Sweet et al. 2006) are among those more frequently used. Most studies conducted in both clinical and healthy populations have shown activations of frontoparietal brain areas involved in attention processing during the aforementioned cognitive tasks (Audoin et al. 2005a, b; Staffen et al. 2002).

However, fMRI studies have also demonstrated significantly altered patterns of cerebral activation in PwMS during the execution of different cognitive tasks (see for a review Chiaravalloti et al. 2015). In particular homologous region adaptation, local activation expansion and extra-region recruitment have been demonstrated to occur in MS. An activation likelihood estimation meta-analytic study (Kollndorfer et al. 2013) showed higher activation in the left ventrolateral prefrontal cortex and right premotor area in PwMS compared to healthy controls. In addition, it has been reported that PwMS can present enlarged patterns of bilateral cortical activations, possibly of compensatory nature (Bonzano et al. 2009; Pantano et al. 2006). Other studies have noted increased task-related fMRI activation in cognitively impaired PwMS related to worse performance on cognitive tasks, suggesting the occurrence of a process of maladaptive plasticity in these patients (Chiaravalloti et al. 2005; Hillary et al. 2003).

Recently, some studies have also provided evidence of improved cognitive performance, as well as improvements in everyday life activities, following cognitive rehabilitation (Amato et al. 2014; Chiaravalloti et al. 2013; Filippi et al. 2012; Mattioli et al. 2010). Among the different cognitive rehabilitation approaches, protocols based on working memory (WM) are of great interest because WM seems to be a mechanism linking cognitive performance to premorbid factors (e.g., verbal intelligence). In fact, WM has been shown to hold a mediating relationship between intellectual enrichment and long-term memory decline in individuals with MS (Sandry and Sumowski 2014). Moreover, WM capacity can be increased by training, as shown by behavioral (Kawashima et al. 2005; Uchida and Kawashima 2008) and neuroimaging (Olesen et al. 2004; Takeuchi et al. 2010) studies. In this regard, we have recently developed an application software for portable devices, named Cognitive Training Kit (COGNI-TRAcK), able to administer user-friendly and personalized treatments based on WM exercises that allow at-home interventions (Tacchino et al. 2015). Using this tool, we then evaluated the efficacy of adaptive (i.e., with fine tuning of exercises difficulty levels to the individual’s performance) vs. non-adaptive WM training in cognitively impaired PwMS treated by means of COGNI-TRAcK (Pedullà et al. 2016). Our results demonstrated that the former approach has better outcomes, showing a significant performance improvement after the intervention only in the adaptive group even in tests evaluating non-trained cognitive domains, with positive effect maintained also after six months.

Based on these findings, we hypothesized that adaptive WM training would also induce changes in functional brain activation related to cognitive performance.

In this study, we aimed to test such hypothesis investigating the effects of adaptive WM training on functional brain activation in PwMS. To achieve this goal, we examined possible changes in brain activation obtained during a cognitive task before and after the training in a group of PwMS, compared to the brain activation pattern elicited by the same task in a group of healthy subjects (included here as a “reference pattern”). Specifically, a group of PwMS underwent a home-based treatment by means of COGNI-TRAcK, with automatic adjustment of tasks difficulty to individual’s performance. Before and after the treatment, cognitive status was assessed using a validated MS-specific neuropsychological battery including PASAT. Brain activity was investigated by fMRI during the PVSAT. Healthy subjects underwent the same fMRI procedure once, and their cognitive performance was assessed with PASAT.

We expected to find alterations in task-related functional activation in PwMS at baseline, including an increase in the extent of activation of the brain areas used by healthy subjects as well as a recruitment of additional brain areas. We also hypothesized that the treatment might induce modifications in brain activity patterns, also based on brain efficiency in recruitment of resources, related to cognitive performance improvements.

## Materials and methods

### Subjects

The sample of this study was constituted of eighteen PwMS. The subjects were recruited among those complaining for cognitive disturbances during formal medical examination performed by a physician of the Italian Multiple Sclerosis Association rehabilitative center of Genoa.

The Rao’s Brief Repeatable Battery of Neuropsychological Tests (BRB-NT) (Rao and The Cognitive Function Study Group of the National Multiple Sclerosis Association 1990) was used to assess cognitive functions at baseline.

Only patients who failed at least two tests of the BRB-NT, i.e., scoring 1.5 standard deviations (SD) below the normative values provided by Amato et al., 2006a, b, were considered cognitively impaired (Amato et al. 2010; Dackovic et al. 2016) and recruited for this study.

The BRB-NT assesses the most frequently impaired cognitive domains in PwMS and incorporates the following tests: Selective Reminding Test, for verbal memory acquisition (Selective Reminding Test-Long-Term Storage, SRT-LTS; Selective Reminding Test-Consistent Long Term Retrieval, SRT-CLTR) and delayed recall (Delayed Selective Reminding Test-Delayed, SRT-D); 10/36 Spatial Recall Test (SPART), for visual memory acquisition and delayed recall (Selective Reminding Test-Delayed, SRT-D); Paced Auditory Serial Addition Test in its variations at different stimuli presentation speed (PASAT-3 and PASAT-2) and Symbol Digit Modalities Test (SDMT), for sustained attention, concentration and information processing speed. We also added the Word List Generation (WLG) test for verbal fluency on semantic stimulus. The tests score was adjusted for years of formal education following the methodology described by Amato et al. 2006. The adjusted scores were used for all the analyses conducted in this study.

All the patients included in this study did not show any relapse in the 3 months prior enrolment nor were under corticosteroids treatments in that same time period. None of the subjects had positive history for major psychiatric disorders or for major medical comorbidities. Moreover, participants were not assuming benzodiazepines nor antidepressants during the study period, and did not present severe visual loss, dyscalculia, acalculia or MRI contraindication.

Among the enrolled patients (12 females and 6 males; mean age: 45.3 ± 10.2 years; mean education: 12.5 ± 3.1 years), 12 were affected by a relapsing-remitting and 6 by a secondary-progressive MS course. In addition, 11 out of 18 patients were treated with disease-modifying drugs. The mean disease duration was 14.3 ± 8.9 years and median Expanded Disability Status Scale (EDSS) (Kurtzke 1983) score was 3.5, range: 1.0–6.5. In 12 out of 18 patients fatigue was perceived as a significantly impacting symptom, since they reported a score > 38 at the Modified Fatigue Impact Scale (MFIS: mean score = 48.1 ± 15.3, range = 16–75) (Flachenecker et al. 2002).

We also recruited eighteen age- and sex-matched healthy subjects (HS) as a control group (10 females and 8 males; age: mean = 41.6 ± 1.3 years, range: 38–43 years). HS were not suffering from neurological or psychiatric disorder nor had personal or familiar history of mood disorders or substance abuse and showed no MRI contraindications.

The study was approved by the Ethics Committee of Azienda Ospedaliera San Martino, Genoa, Italy. All subjects provided a written informed consent according to the Declaration of Helsinki.

### Rehabilitation protocol

The PwMS participating in the study executed an 8-week training consisting of five 30-min sessions a week. The sessions were self-administered at home by means of COGNI-TRAcK, with adaptive algorithms automatically adjusting the progression in task difficulty on the basis of the individual’s performance. Three different types of WM exercises were executed every session (a visuospatial WM task, an “operation” *N*-back task and a “dual” *N*-back task) as described elsewhere (Tacchino et al. 2015). Briefly, in the visuospatial WM task patients had to remember a random sequence of visual stimuli presented one at a time in a grid-like interface and correctly reproduce it by touching the corresponding locations on the screen. In the “operation” *N*-back task, the stimuli consisted of pairs of numbers, 1 to 4, shown in a random sequence on the screen (e.g., 1 + 4). Participants were asked to memorize the sum of the two numbers presented (ranging from 2 to 8) and to push the button on the keyboard that corresponded to *N* stimuli ago (*N*-back rule). In the “dual” *N*-back task, the stimuli consisted of numbers, 1 to 4, presented in a random sequence in one of four places on a line. Patients were asked to memorize the location and identity of the stimuli and their temporal order. Then, they had to push two buttons to indicate the identity *and* the location of the stimulus presented, according to the *N*-back rule. The adaptive paradigm adjusted the difficulty level of the exercises to the subject’s performance in order to be more effective in improving cognitive functions in PwMS. Particularly, the exercises difficulty level increased by one step every time the subject performed an exercise correctly and decreased by one step if the exercise was done incorrectly for three times in a row. In details, based on a previous study (Takeuchi et al. 2010), in the first visuospatial WM task the level of difficulty was varied by changing the number of presented stimuli (minimum level: 4 stimuli). When participants indicated the correct location of all the stimuli in the presented order, it was regarded as a correct answer. In the two *N*-back tasks, the level of difficulty was varied by changing the value of *N* (minimum level: *N* = 0). Blocks were regarded as completed correctly when the participants made errors in less than 20% of the trials in the “operation” *N*-back task and less than 25% in the “dual” *N*-back task. In addition to the paradigm proposed by Takeuchi and colleagues, the inter-stimulus interval was also adjusted according to individual participant’s performance. Thus, every main level, determined by the number of stimuli for the visuospatial WM task and by the *N* parameter for the *N*-back tasks, was constituted by sub-levels with increased inter-stimulus rate. For each type of exercise, all PwMS started from the same low difficulty levels (namely, 2 s for the visuospatial WM task, 5 s for the “operational” *N*-back task and 7 s for the “dual” *N*-back task) in order to allow all patients with cognitive impairment to begin the training (Pedullà et al. 2016).

Adherence to treatment, calculated as the percentage of completed sessions on the total number of scheduled sessions, and mean results of each training session (i.e., difficulty level, accuracy of answers and, only for the *N*-back tasks, reaction time) were measured for each patient.

### Cognitive performance evaluation

Before (“PRE session”, i.e., baseline) and after (“POST session”) the rehabilitation treatment, cognitive performance was evaluated in all PwMS with the BRB-NT in order to detect possible functional changes induced by the intervention. Version A and version B (Goretti et al. 2014) were adopted for the two sessions and randomly assigned so that half of the patients participating in the study received version A first while the other half received version B first. All tests were administered by a trained and certified cognitive examiner blinded to the session phase (POST vs. PRE).

Furthermore, in order to assess the magnitude of these changes, the effect size of POST vs. PRE difference in PwMS cognitive performance was calculated by means of Cohen d method (Cohen 1988) and interpreted according to Sawilowsky descriptors (d = 0.01: very small, d = 0.20: small, d = 0.50: medium, d = 0.80: large, d = 1.20: very large, or d = 2.0: huge) (Sawilowsky 2009). Mean percentage change, calculated as ((POST score – PRE score) / PRE score) × 100, was computed for SDMT and PASAT-3, i.e., the tests investigating information processing speed which was shown to be the cognitive domain most widely affected by MS and the first cognitive deficit to emerge in PwMS (Van Schependom et al. 2015).

In addition, cognitive functions of HS were assessed with PASAT-3 only, in order to have a measure of HS cognitive performance to compare with PwMS performance. Indeed, this test was chosen among those included in the BRB-NT because it is the auditory analogue of PVSAT, that is the cognitive task all subjects (PwMS and HS) had to perform during the fMRI sessions.

### MRI acquisition

MRI was acquired on a 1.5 T MR system (Signa HDxt General Electric, WI, USA) and included the following brain sequences: sagittal T1-weighted 3D spoiled gradient recalled (SPGR) imaging (slice thickness = 1.2 mm; TR = 9.4 ms; TE = 3.9 ms; flip angle = 8, FOV = 240 × 240 mm; matrix = 256 × 256) for atrophy estimation, axial T2-weighted imaging (slice thickness = 5 mm; TR = 4000 ms; TE = 104.6 ms; FOV = 240 × 240 mm; matrix = 256 × 256) as reference for fMRI, and axial single-shot spin-echo echoplanar imaging for fMRI (slice thickness = 5 mm; TR = 3000 ms; TE = 60 ms; FOV = FOV = 240 × 240 mm; matrix = 64 × 64). For each subject, the first 3 volumes of each fMRI run were discarded because of non-steady magnetization, and the remaining 80 volumes were used for the analysis. Within each run the subject performed either the PVSAT (i.e., active task) or a baseline visual task (i.e., control), according to a boxcar paradigm with two 30-s active task periods alternating with two 30-s control periods (Bonzano et al. 2009). During the PVSAT phase, Arabic digits (black numbers on a white background, range: 1–9) were presented via a projection mirror system one at a time and the subject was instructed to consecutively add pairs of numbers, such that each number was added consecutively to the one that immediately preceded it. The baseline visual task consisted of the presentation of a fixation cross. In both phases, each stimulus was presented for 1 s and followed by a blank screen for 2 s. All participants familiarized with the PVSAT and the experimental protocol outside the scanner before the beginning of the imaging session. While inside the scanner they were instructed to calculate and not to spell the numbers or results to avoid artifactual brain activations. At the end of the fMRI session, they were asked to report the last sum obtained. As for PASAT, we adopted two different series of numbers randomly assigned to the subjects for the two experimental sessions.

### Structural MRI analysis

For each patient and session, normalized brain volume (NBV) was estimated from T1-weighted 3D SPGR images with SIENAX (Smith et al. 2002). Segmentation of brain, including cerebellum, from non-brain tissue in the head was performed. The outer skull surface was estimated to normalize brain volume for skull size; all brain and skull images were registered to a standard space and a normalized brain volume estimate was obtained.

### Functional MRI analysis

fMRI data processing was performed with Statistical Parametric Mapping software (SPM12, Wellcome Department of Imaging Neuroscience, London, UK) as described elsewhere (Friston et al. 1995). Briefly, each subject’s time series were movement-corrected, normalized to the Montreal Neurological Institute (MNI) template brain image using a 12-parameter affine transformation and smoothed using an 8 mm full-width at half-maximum isotropic Gaussian kernel to increase the signal-to-noise ratio.

### Statistical analysis

#### Cognitive training and performance

Changes in the training execution were investigated using an analysis of variance analysis (ANOVA) on mean difficulty level and accuracy of each session with TIME as within-subject factor (sessions 1 to 40) for all three types of exercises. Mean reaction time of each session was investigated only for the *N*-back tasks. Missing data of non-completed sessions were filled up with the values of the previous session, according to the “last observation carried forward” analysis (Altman 2009).

Concerning cognitive performance, data distribution was explored with the Shapiro–Wilk test to assess each BRB-NT test’s score for normality. A paired t-test was used for each test of the battery in order to detect significant changes between the two sessions (PRE and POST) in the group of PwMS. Moreover, the PASAT-3 scores of PwMS in the two sessions were compared with HS performance by means of a t-test for independent samples.

#### fMRI analysis

A general linear model was used to identify the voxels with task-related signal changes at the individual level. Task-related t contrast images were created for every participant and were then introduced into a second-level random-effect analysis to allow for population inferences. One-sample t-tests were adopted to obtain the group activation maps (HS, PwMS in the PRE and POST sessions), with a height threshold of *p* < 0.05 FWE-corrected and an extent threshold of 30 voxels per cluster. A paired t-test was then used to assess differences between the two sessions (PRE and POST) in the group of PwMS and t-tests for independent samples were used to assess differences in the PwMS in the single sessions separately with respect to HS. The analyses of statistical contrasts between sessions and groups were conducted with height threshold of *p* < 0.001 uncorrected and minimum cluster size of 30 voxels.

#### Correlation analysis

Following the contrast analysis “PRE session > POST session” we found significant clusters located in the left cingulate gyrus (BA 31), the right postcentral gyrus and inferior parietal lobule (BA 40). However, the right BA 40 was still significantly active in the POST session. To shed light on the role of this brain region on cognitive task performance, the first eigenvariate of the blood-oxygenation-level dependent (BOLD) fMRI signal was extracted in the corresponding significant cluster and correlated with PASAT performance adjusting for the NBV for the values obtained in the PRE and in the POST session, respectively.

## Results

### Cognitive training and performance

All patients completed the proposed protocol training along the 8 weeks without reporting any complaint and with a high adherence to treatment rate in all three exercise types (89 ± 13%, 87 ± 13% and 85 ± 13% for the visuospatial WM task, “operation” and “dual” *N*-back task, respectively).

**Fig. 1:**
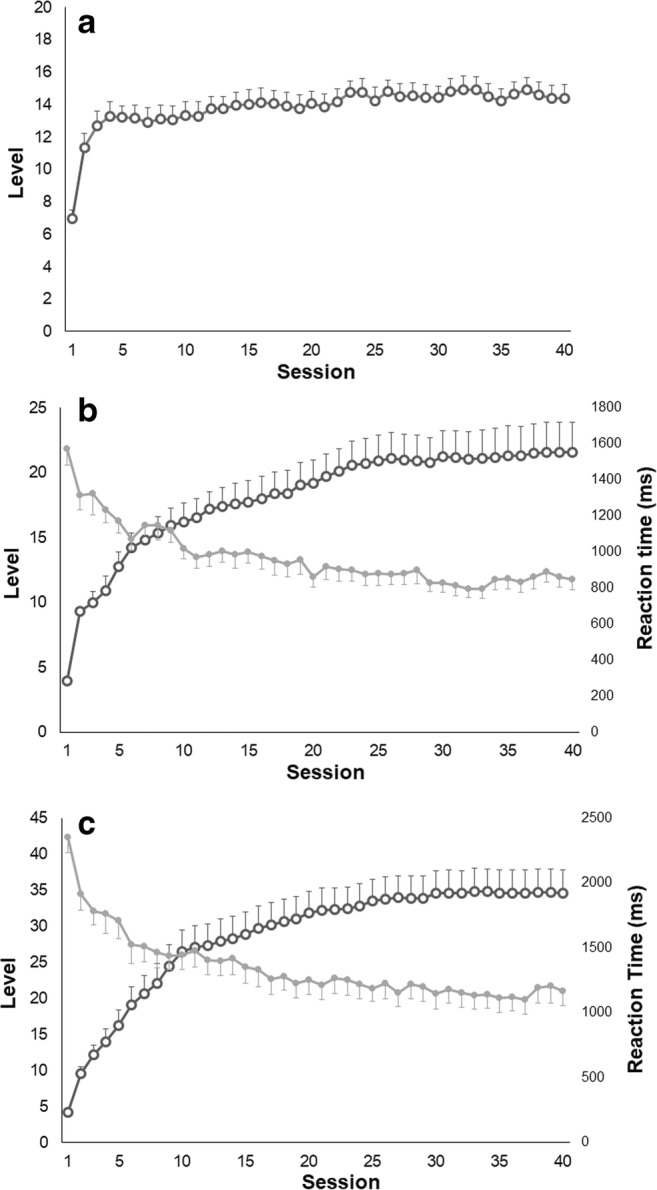
Data showing the mean performance of subjects during the cognitive training: **a** mean difficulty level reached in each session in the visuospatial WM task; **b** mean difficulty level and reaction time of each session in the “operation” *N*-back task; **c** mean difficulty level and reaction time of each session in the “dual” *N*-back task. Error bars represent standard errors

Concerning the visuospatial WM task, the ANOVA showed a significant increase of the difficulty level reached throughout the 40 sessions (F_39,663_ = 18.34, *p* < 0.0001) (Fig. 1a). Accuracy changed (F_39,663_ = 3.11, p < 0.0001), but only between the first and the other sessions of the treatment, with higher values obtained in the first session due to the low starting difficulty levels. With regard to the “operation” *N*-back task, an increase of the difficulty level (F_39,663_ = 19.24, *p* < 0.0001) and a decrease of the reaction time (F_39,663_ = 12.84, p < 0.0001) were observed throughout the treatment (Fig. 1b). Again, accuracy decreased (F_39,663_ = 2.86, *p* < 0.0001) only between the first and the other sessions of the training. Similar results were found concerning the “dual” *N*-back task (Fig. 1c): the difficulty level increased throughout the 40 sessions (F_39,663_ = 36.17, *p* < 0.0001), whilst the reaction time decreased with TIME (F_39,663_ = 19.91, p < 0.0001). In this case, accuracy changed slightly (F_39,663_ = 1.45, *p* = 0.04), decreasing between the first and the following sessions.

At baseline, all subjects obtained a score lower than 1.5 SD below the normative data at two or more tests composing the BRB-NT. The median number of failed tests was 4 (range 2–8), with highest prevalence of failing observed at the SRT-LTS (72% of patients), followed by SRT-CLTR (67%), SRT-D and PASAT-2 (61%). Half of the patients failed at PASAT-3 and SDMT, whilst lower percentages were observed at SPART (44%) and SPART-D (17%). None of the patients failed at the WLG.Shapiro-Wilk test showed that all tests’ results were normally distributed, allowing the use of parametric statistics.

The WM training was effective in improving the cognitive status of PwMS, as shown by their performance at the BRB-NT. In fact, PwMS showed significantly higher scores at all the tests in the POST session than in the PRE session (Table 1). Effect size was large or very large for all tests (SRT-LTS: d = 1.64, SRT-CLTR: d = 1.31, SPART: d = 1.03, PASAT-3: d = 1.34, PASAT-2: d = 1.48, SRT-D: d = 1.84, SPART-D: d = 1.03, WLG: d = 0.99), except for SDMT which showed medium effect size (d = 0.76). Mean percentage POST vs. PRE change was 20.73% for SDMT and 60.01% for PASAT-3.

**Table 1:** Scores obtained by PwMS at the BRB-NT before (PRE) and after (POST) the rehabilitative intervention

Test	PRE mean (SD)	POST mean (SD)	*p* value	t
SRT LTS	24.74 (7.51)	41.35 (12.25)	0.000003	6.80
SRT CLTR	16.18 (8.91)	33.03 (15.81)	0.000184	4.75
SPART	14.46 (4.25)	19.27 (5.07)	0.001351	3.83
SDMT	39.96 (10.33)	48.24 (11.40)	0.000008	6.28
PASAT-3	27.70 (12.77)	44.32 (12.06)	0.000000	8.16
PASAT-2	18.97 (9.02)	33.02 (9.99)	0.000006	6.48
SRT-D	5.43 (1.72)	8.97 (2.12)	0.000004	6.62
SPART-D	4.56 (1.08)	6.31 (2.13)	0.003348	3.41
WLG	38.94 (5.72)	46.89 (9.78)	0.000063	5.26

Student’s t test values are reported

*SRT-LTS*, Selective Reminding Test-Long Term Storage; *SRT-CLTR*, Selective Reminding Test-Consistent Long-Term Retrieval; *SPART*, 10/36 Spatial Recall Test; *SDMT*, Symbol Digit Modalities Test; *PASAT-3/−2*, Paced Auditory Serial Addition Test; *SRT-D*, Selective Reminding Test-Delayed; *SPART-D*, 10/36 Spatial Recall Test-Delayed; *WLG*, Word List Generation

### Brain activation during PVSAT

**Fig. 2:**
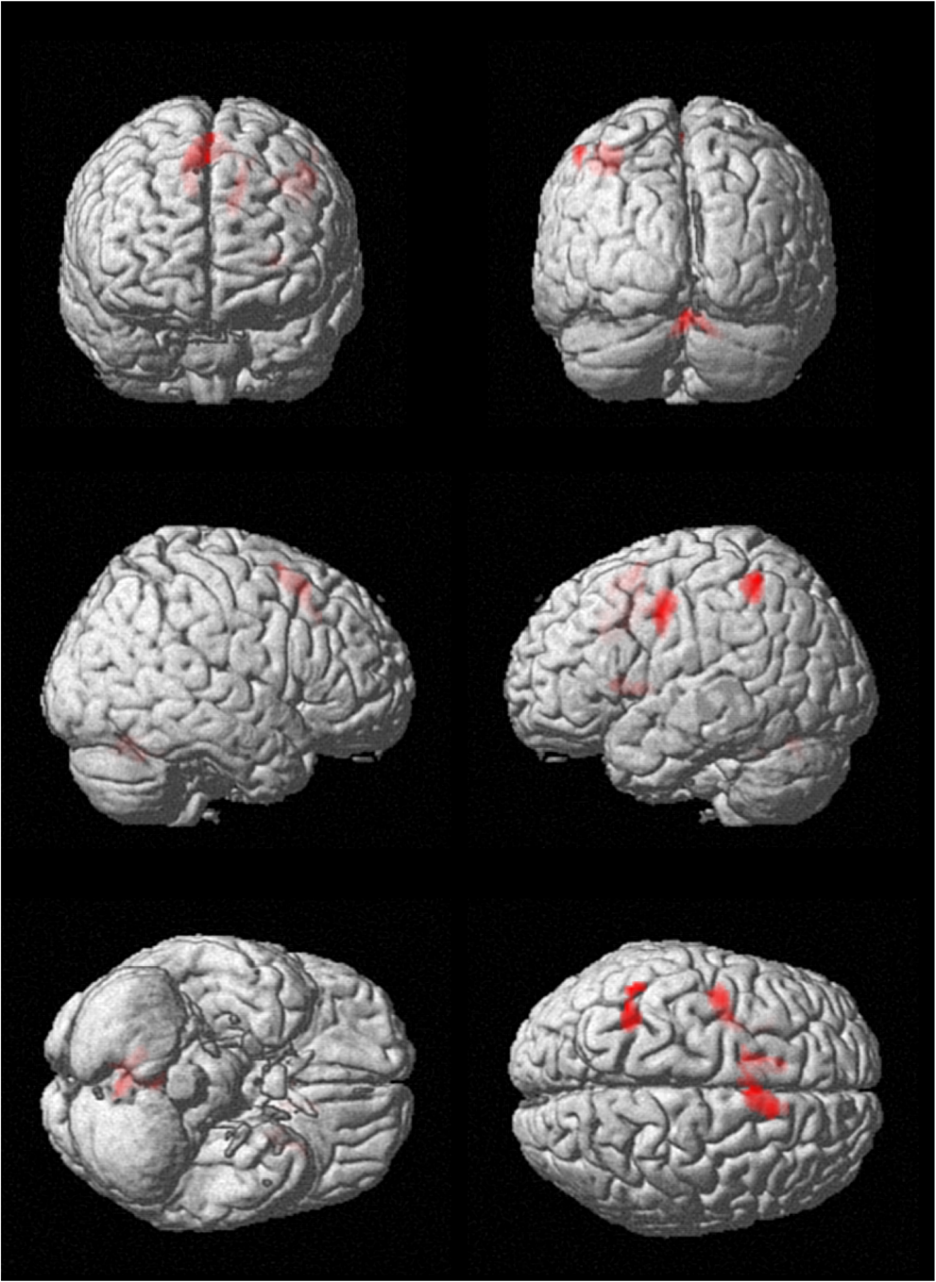
Group activation map of healthy subjects during the PVSAT (see Table 2 for details). Significant blobs are displayed on a rendering surface (height threshold *p* < 0.05 FWE-corrected; extent threshold k = 30 voxels)

**Fig. 3:**
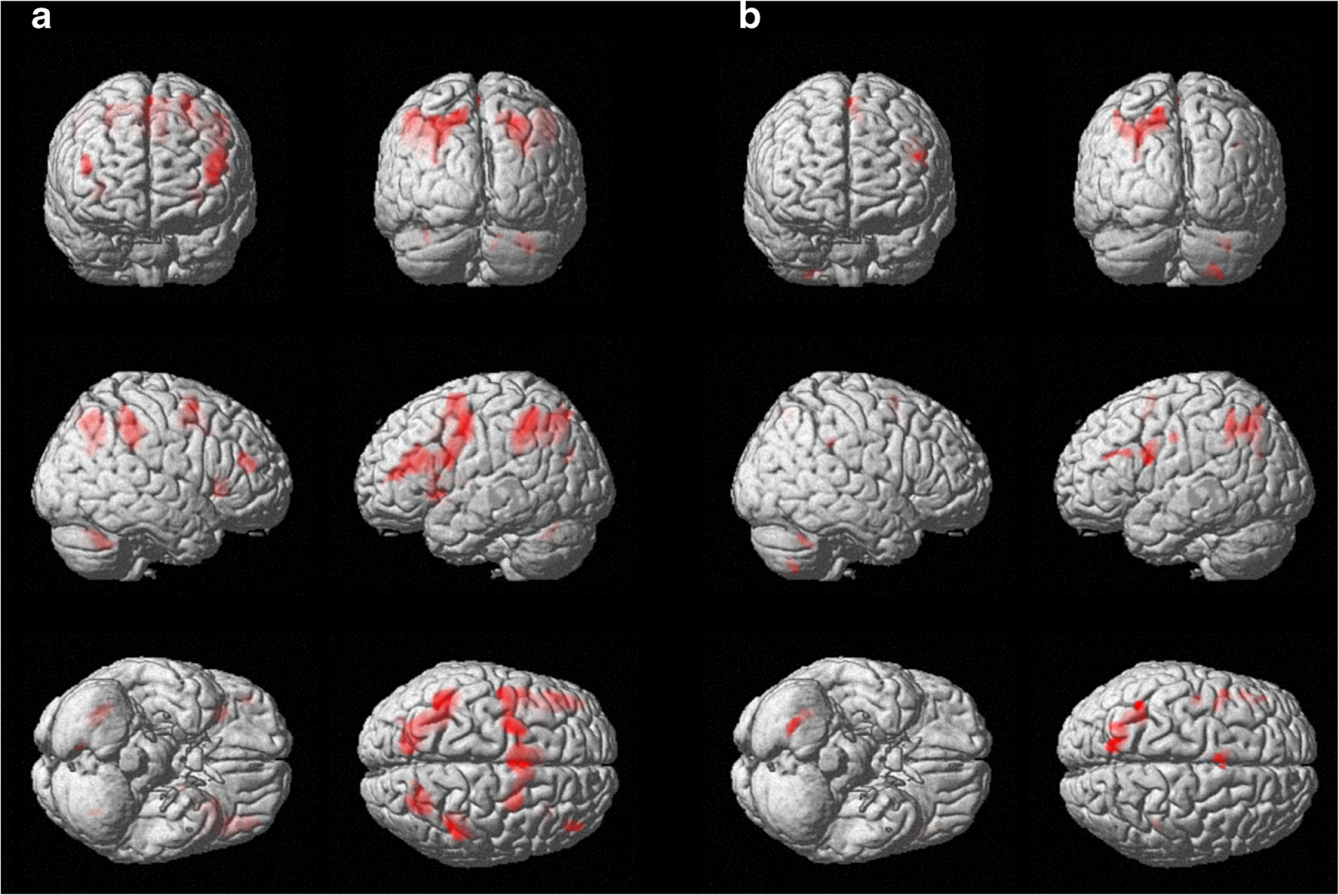
Group activation blobs displayed on a rendering surface for the PwMS group (height threshold p < 0.05 FWE-corrected; extent threshold k = 30 voxels) in the two experimental sessions: **a** before the rehabilitative treatment, **b** after the rehabilitative treatment (see Table 2 for details)

The coordinates of the significant peaks of activation associated to PVSAT in the different groups are reported in Table 2. As also displayed in Fig. 2, the group activation map obtained for HS primarily included clusters located in the left cingulate gyrus (Brodmann’s area (BA) 32), precuneus (BA 7), inferior parietal lobule (BA 40), precentral and middle frontal gyri (BA 6), and in the right cerebellum. The pattern of activation obtained in the PwMS group before cognitive rehabilitation (PRE session) includes bilateral activation clusters in the precuneus (BA 7), inferior parietal lobule (BA 40), and cerebellum, and clusters in the left BA 6, and in the right postcentral gyrus (BA 2) and insula (BA 13) (Fig. 3a).

**Table 2:** Brain regions significantly activated during the PVSAT for the different groups and sessions (height threshold of p < 0.05 FWE-corrected, extent threshold of 30 voxels)

Group	Cluster size	Voxel T	Voxel Z	MNI Coordinate: x y z (mm)	Laterality	Anatomical Location	Brodmann’s Area
HS	577	13.02	6.31	12 20 52	Right	Cingulate Gyrus	32
		11.64	6.03	−12 26 32	Left	Cingulate Gyrus	32
		9.55	5.54	−14 10 42	Left	Cingulate Gyrus	32
	199	10.33	5.74	−30 -48 54	Left	Precuneus	7
		8.19	5.15	−46 − 48 56	Left	Inferior Parietal Lobule	40
		7.58	4.95	-48 -48 48	Left	Inferior Parietal Lobule	40
	285	10.1	5.68	−42 -4 38	Left	Precentral Gyrus	6
		8.94	5.37	−32 0 42	Left	Middle Frontal Gyrus	6
		8.74	5.31	−42 -8 46	Left	Precentral Gyrus	6
	198	9	5.39	0–68 − 24	Right	Cerebellum	
		8.22	5.16	14–58 -28	Right	Cerebellum	
		8.05	5.1	0–52 -26	Right	Cerebellum	
	113	8.52	5.25	-24 6 4	Left	Putamen	
		7.92	5.06	−30 16 6	Left	Claustrum	
PwMS (PRE)	3818	15.02	6.65	−26 0 58	Left	Sub-Gyral	6
		14.08	6.49	−6 10 48	Left	Medial Frontal Gyrus	32
		13.09	6.32	−48 8 22	Left	Inferior Frontal Gyrus	44
	1894	12.25	6.16	−30 -48 36	Left	Inferior Parietal Lobule	40
		11.77	6.06	−42 -50 48	Left	Inferior Parietal Lobule	40
		10.91	5.87	−14 -72 52	Left	Precuneus	7
	750	12	6.11	28–64 40	Right	Superior Parietal Lobule	7
		7.54	4.93	14–68 50	Right	Precuneus	7
	232	10.45	5.77	34–60 -36	Right	Cerebellum	
	642	10.34	5.74	48–36 44	Right	Inferior Parietal Lobule	40
		10.09	5.68	36–36 32	Right	Postcentral Gyrus	2
		9.18	5.44	40–42 44	Right	Inferior Parietal Lobule	40
	188	9.4	5.5	34 22 0	Right	Insula	13
		9.34	5.48	32 22 8	Right	Insula	13
	48	8.91	5.36	−30 -78 26	Left	Superior Occipital Gyrus	39
	117	8.87	5.35	44 46 18	Right	Middle Frontal Gyrus	10
	51	7.7	4.99	−34 -60 -30	Left	Cerebellum	
	32	7.33	4.86	10–72 -34	Right	Cerebellum	
PwMS (POST)	752	11.44	5.99	−26 -64 40	Left	Superior Parietal Lobule	7
		10.69	5.82	−16 -62 46	Left	Precuneus	7
		9.51	5.53	−10 -66 50	Left	Precuneus	7
	65	9.92	5.64	26–64 -52	Right	Cerebellum	
	42	9.61	5.55	−42 -10 36	Left	Precentral Gyrus	6
	63	9.41	5.5	34–58 -34	Right	Cerebellum	
	186	8.53	5.25	−50 8 22	Left	Inferior Frontal Gyrus	44
		8.19	5.15	−40 4 26	Left	Inferior Frontal Gyrus	9
	33	8.35	5.2	42–38 32	Right	Inferior Parietal Lobule	40
	118	8.24	5.16	−6 8 52	Left	Medial Frontal Gyrus	32
		7.87	5.04	−2 6 62	Left	Superior Frontal Gyrus	6
	59	8.07	5.11	−48 22 26	Left	Middle Frontal Gyrus	46
		7.96	5.07	−46 36 24	Left	Middle Frontal Gyrus	46

After cognitive rehabilitation (POST session), PwMS showed a different activation map (Fig. 3b), with clusters mainly located in the right cerebellum and in the left hemisphere: precuneus and superior parietal lobule (BA 7), precentral and superior frontal gyri (BA 6). The only significant cluster in the right hemisphere was located in the inferior parietal lobule (BA 40).

As reported in Table 3, the contrast analysis between sessions (PRE and POST) in the PwMS group showed that the left cingulate gyrus (BA 31), the right postcentral gyrus and the right inferior parietal lobule (BA 40) were significantly more active in the PRE session compared to the POST session. The opposite contrast (POST vs. PRE) gave no suprathreshold clusters.

**Table 3:** Brain regions showing significantly different activation during the PVSAT in the PwMS group between the two sessions (height threshold of p < 0.001 uncorrected, extent threshold of 30 voxels)

Contrast	Cluster Size	Voxel T	Voxel Z	MNI Coordinate: x y z (mm)	Laterality	Anatomical Location	Brodmann’s Area
PwMS (PRE) - PwMS (POST)	41	4.8	3.76	−12 -26 38	Left	Cingulate Gyrus	31
	36	4.14	3.4	48–36 52	Right	Postcentral Gyrus	40
		3.94	3.27	56–34 48	Right	Inferior Parietal Lobule	40
PwMS (POST) - PwMS (PRE)	No suprathreshold clusters						

### Correlation between brain activity and task performance

As we found that the left BA31 and the right BA40 significantly reduced their activation after the training but only the right BA40 was still significantly active in the POST session, we extracted the first eigenvariate of the BOLD signal in the cluster located in the right BA 40 in order to identify any possible relationship of this area with cognitive performance.

**Fig. 4:**
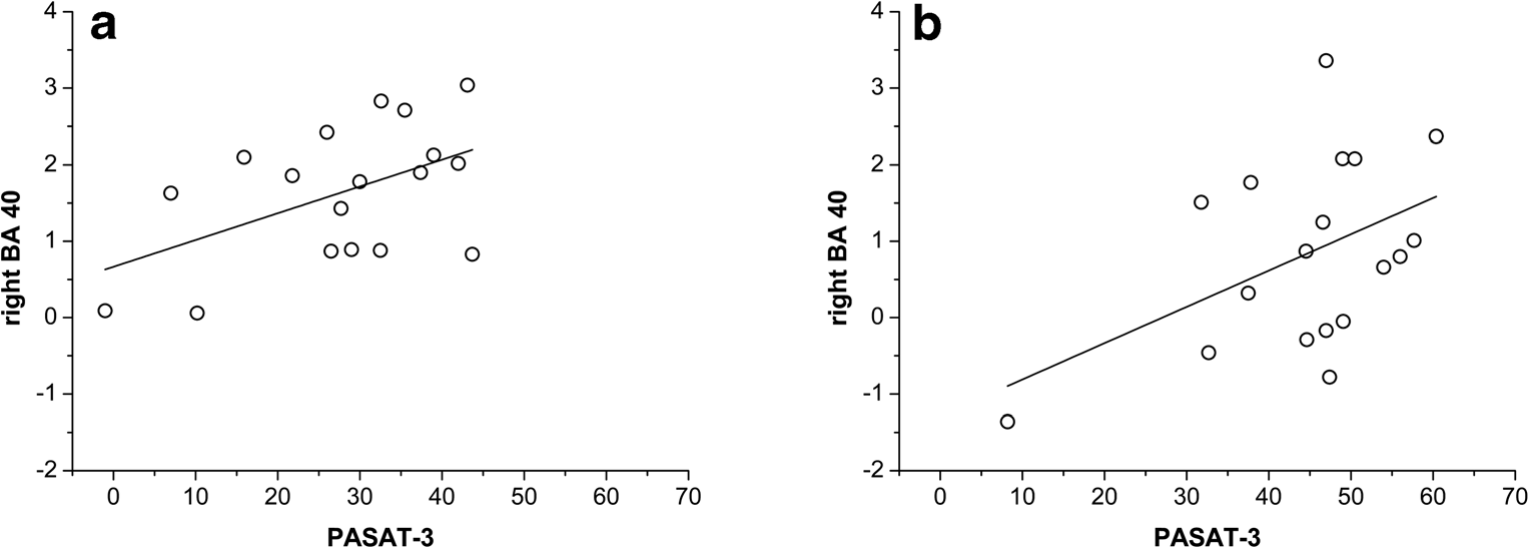
Linear relationship between PASAT-3 and the first eigenvariate of the BOLD signal in the right BA 40 before (**a**) and after (**b**) the cognitive rehabilitation treatment

Both in the PRE and in the POST sessions a significant correlation was found between the PASAT-3 and the BOLD signal in the right BA 40 (PRE: *r* = 0.51, *p* = 0.02; POST: *r* = 0.46, *p* = 0.03), indicating that patients with better performance had a higher activation of this brain region both before and after the rehabilitation treatment (Fig. 4). This relationship was confirmed when applying partial correlations to adjust for NBV (right BA 40 PRE: *r* = 0.50, p = 0.02; POST: *r* = 0.48, p = 0.03).

### Comparison of PwMS to HS

**Fig. 5:**
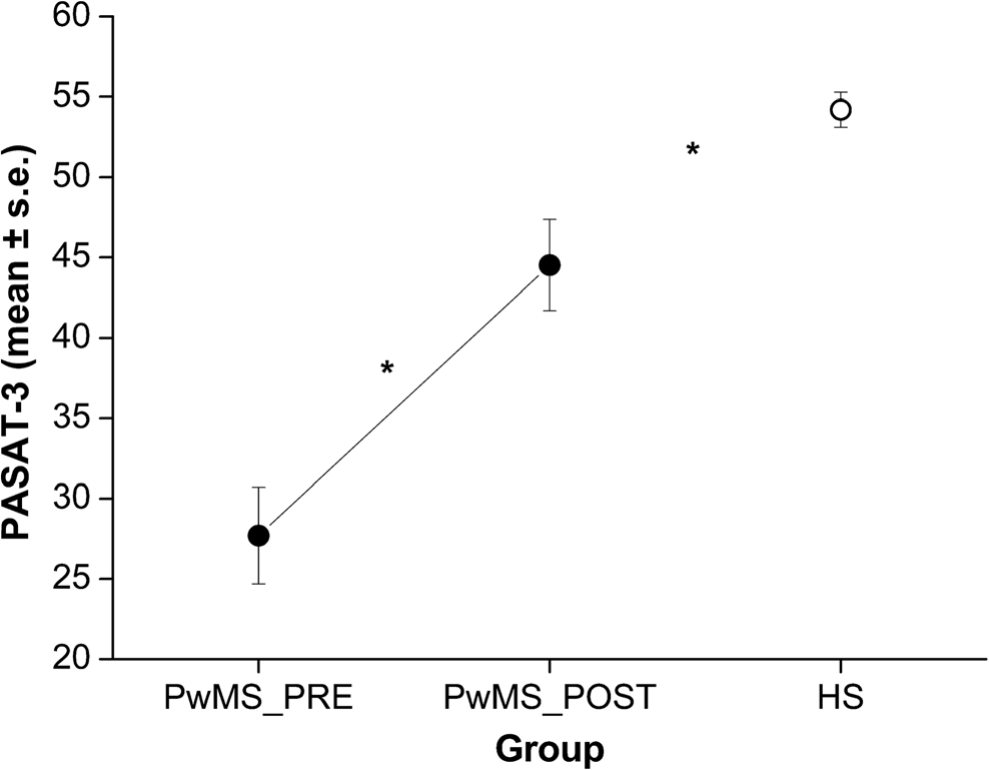
PASAT-3 corrected scores of the PwMS group, before (PRE) and after (POST) the rehabilitative intervention, and of the group of healthy subjects (HS). * indicate *p* < 0.05 (PwMS_POST vs. PwMS_PRE and PwMS_post vs. HS)

PwMS showed worse performance at PASAT-3 than HS both before (*t* = 8.28, *p* < 0.001) and after (*t* = 3.18, *p* = 0.003) the rehabilitative intervention (Fig. 5).

Concerning the fMRI activity the results from the contrast analysis to compare PVSAT-related activation between PwMS and HS revealed that PwMS significantly activated more clusters in both hemispheres in the PRE session (See Table 4). In the POST session, PwMS showed increased activation in some clusters located in the left frontoparietal regions compared to HS.

**Table 4:** Brain regions showing significantly different activation during the PVSAT between groups (height threshold of p < 0.001 uncorrected, extent threshold of 30 voxels)

Contrast	Cluster Size	Voxel T	Voxel Z	MNI Coordinate: x y z (mm)	Laterality	Anatomical Location	Brodmann’s Area
PwMS (PRE) - HS	423	5.70	4.74	−8 12 36	Left	Cingulate Gyrus	24
		3.97	3.57	−12 4 54	Left	Medial Frontal Gyrus	6
		3.65	3.33	−20 16 52	Left	Superior Frontal Gyrus	8
	696	5.69	4.73	−40 42 12	Left	Middle Frontal Gyrus	10
		5.02	4.31	−48 28 16	Left	Inferior Frontal Gyrus	46
		4.74	4.12	−44 20 18	Left	Inferior Frontal Gyrus	45
	871	5.52	4.63	−42 -52 50	Left	Inferior Parietal Lobule	40
		4.16	3.71	−60 -40 38	Left	Inferior Parietal Lobule	40
	532	5.46	4.60	−32 -2 40	Left	Precentral Gyrus	6
		4.76	4.14	−40 -6 40	Left	Precentral Gyrus	6
		4.20	3.74	−34 -2 24	Left	Insula	13
	143	4.79	4.16	30 24 4	Right	Insula	13
	275	4.52	3.97	−22 -76 44	Left	Superior Parietal Lobule	7
		4.04	3.63	−12 -76 50	Left	Precuneus	7
	326	4.40	3.88	56–28 34	Right	Inferior Parietal Lobule	40
		4.20	3.74	42–34 28	Right	Inferior Parietal Lobule	40
		3.59	3.28	64–26 38	Right	Postcentral Gyrus	2
	67	4.38	3.87	28–68 44	Right	Superior Parietal Lobule	7
	43	4.29	3.81	−28 16 2	Left	Claustrum	
	38	4.01	3.60	36 6 22	Right	Insula	13
	72	3.91	3.53	−10 -60 38	Left	Precuneus	7
		3.65	3.33	−4 -68 36	Left	Precuneus	7
HS - PwMS (PRE)	No suprathreshold clusters						
PwMS (POST) - HS	122	4.48	3.94	−36 42 12	Left	Middle Frontal Gyrus	10
		4.01	3.6	−44 38 14	Left	Inferior Frontal Gyrus	46
	37	4.13	3.69	−12 14 32	Left	Cingulate Gyrus	24
	45	3.95	3.56	−8 -62 38	Left	Precuneus	7
	46	3.92	3.53	−32 -2 36	Left	Precentral Gyrus	6
HS - PwMS (POST)	60	4.02	3.61	40–24 6	Right	Superior Temporal Gyrus	13

## Discussion

In the present work, we showed that an adaptive training based on working memory improves cognitive performance in PwMS and impacts brain functional activation. Although both in PRE and POST sessions the patients’ performance at PASAT-3 was lower than HS, it significantly increased after training. Moreover, at POST session PVSAT elicited in PwMS a brain activation map which was more similar to that one found in healthy participants compared to the PRE session.

The proposed rehabilitative intervention was very well received by all PwMS, in line with a previous study investigating the usability of COGNI-TRAcK (Tacchino et al. 2015). This result could be related to the advantages of a system allowing an at-home treatment, which avoids frequent travels to the rehabilitation center.

The improvement in cognitive performance observed following the proposed adaptive training based on WM is in line with other studies investigating the effects of cognitive rehabilitation in MS (Amato et al. 2014; Chiaravalloti et al. 2013; Filippi et al. 2012; Mattioli et al. 2010; Pedullà et al. 2016). The adaptive algorithm allowed the patients to train at their maximal working load throughout the whole amount of the intervention, as shown by the increase of the difficulty levels in spite of a constant accuracy percentage. It is worth noting that in the *N*-back tasks reaction time decreased from the first to the last session of the training although the difficulty of the exercises increased. This is an important result since previous research showed that PwMS responded significantly slower than controls on reaction time tests and that the difference between the performances of the two groups progressively increased as the tests became more difficult, i.e. as processing demands increased (Reicker et al. 2007). In our study participants improved their speed of processing ability similarly to a sample of PwMS who underwent a specific computer based home training of divided attention that aimed to improve this cognitive function (Pusswald et al. 2014). Thus, we may suggest that the proposed adaptive training based on WM has a positive effect on trained (e.g., information processing speed, sustained attention and visual memory) and non-trained (e.g., alertness, divided attention, verbal memory and fluency) cognitive domains, corroborating previous evidence supporting a mediating/intervening role of WM in other cognitive functions in PwMS (Berrigan et al. 2013).

Importantly, intervention-associated changes were not only statistically significant, but also meaningful in terms of magnitude of the difference, as shown by the effect size analysis. Moreover, improvements in SDMT and PASAT-3 were widely above the percentage accepted in the literature as minimally clinically relevant difference (reported as 10% for SDMT (Benedict et al. 2017) and 20% for PASAT-3 (Hoogervorst et al. 2004)).

The lack of a control group of PwMS does not allow disentangling between training effects and other unspecific effects such as time or the repetition of assessment. However, in order to minimize form-specific practice effects, equivalent forms were used and participants were randomly assigned to receive Form A or B in the PRE session and then switched in the POST session. Thus, we can suggest that the improvement in cognitive performance in PwMS measured in this study was a direct effect of the treatment.

In parallel to cognitive performance improvement, we found a significant reduction of cortical activity after COGNI-TRAcK treatment. In particular, contrast analysis identified the left cingulate gyrus and the right inferior parietal lobule (BA40) as the areas where activity significantly reduced after the intervention.

Conversely, other authors have shown increased activation of existing networks underlying trained functions in PwMS following cognitive rehabilitation (Chiaravalloti et al. 2012; Ernst et al. 2012). Neuroplasticity of cognitive functions in PwMS can occur thanks to different neural processes concerning homologous region adaptation, local activation expansion, and extra-region recruitment towards the maintenance of cognitive functioning (Chiaravalloti et al. 2015). In our study, we found abnormal brain activation during PVSAT compared to healthy subjects before treatment, with a pattern of activation including several bilateral activation clusters. In cross-sectional studies it is generally not easy to differentiate the adaptive components of plasticity due to functional architecture response to tissue damage from maladaptive changes that negatively impact cognitive performance. Here, however, thanks to our longitudinal design we observed that improvement in cognitive performance over time was associated with a size reduction of the activation pattern thus indirectly suggesting a recovery from a possible condition of maladaptive neuroplasticity. Maladaptive neuroplasticity may come at the cost of other cognitive functions. Therefore, effective cognitive rehabilitation programs should be aimed at inducing adaptive neuroplasticity, “normalizing” brain function and behavioral output. Following our cognitive rehabilitation program, PwMS showed reduced task-related activation with clusters mainly located in the right cerebellum and in the left-brain hemisphere; the only significant cluster in the right hemisphere was located in the inferior parietal lobule (BA 40).

These findings may imply that WM training can improve the cognitive performance and reduce the abnormal activation of cognitively impaired PwMS, partially maintaining or even restoring the brain cognitive function of PwMS and restricting the neural recruitment to the only brain areas that are responsible for the maintenance of function (greater “brain efficiency”). Especially, we could suggest that adaptive training based on WM could be extremely useful because of its ability to tune step-by-step performance. Indeed, lowering the level of tasks difficulty in the case of unsuccessful performance might reduce the stress encountered by the individual. On the other hand, the tailored difficulty increase consequential to patient improvement might physiologically activate existing networks for cognitive functioning stimulating a more accurate compensatory adaptive process (Voytek et al. 2010).

The right BA40 could represent a basic compensatory area whose activation may help coping for brain damage in PwMS in order to sustain attentional tasks. This hypothesis is strongly supported by the positive partial correlation between the PASAT-3 scores and the right BA40 BOLD signal when controlling for brain atrophy (here expressed as NBV), which was significant both at PRE and at POST sessions.

The right inferior parietal lobule has been shown to be involved both in mathematical processing (Chochon et al. 1999) and attentional resources allocation (Singh-Curry and Husain 2009), which represent two of the key components underlying PVSAT performance. In line with these findings, a crucial role of the inferior parietal lobule in working memory and sustained attention tasks, such as the PVSAT, was observed in a recent meta-analysis conducted on both healthy controls and PwMS (Kollndorfer et al. 2013). The inferior parietal lobule has been demonstrated to be more active during the 2-back task in PwMS with preserved cognition compared to those with cognitive deficits (Rocca et al. 2010) and to present increased functional connectivity with the default mode network after cognitive rehabilitation (Bonavita et al. 2015). Moreover, a resting-state investigation involving PwMS showed increased functional connectivity of the right inferior parietal lobule after cognitive treatment (Parisi et al. 2014). The functional connectivity between the anterior cingulate cortex and the right BA40 was also found to correlate with improvement of PASAT performance, suggesting the occurrence of compensatory mechanisms after cognitive training.

In particular, the right inferior parietal lobule could have a role in cognitive reserve (CR), referred to as the brain active attempt to cope with brain damage using pre-existing cognitive processes or enlisting compensatory strategies (Stern 2002). Indeed, using both fMRI and PET imaging, the inferior parietal lobule has been shown in healthy subjects to be included in a frontoparietal network modulated by CR (Stern et al. 2003, 2005). The CR model could explain the lack of a direct relationship between the extent of brain pathology and the clinical manifestation of cognitive impairment in MS (Benedict et al. 2010; Sumowski et al. 2009).

In this scenario, there is increasing interest for applications able to build cognitive reserve and thus to preserve (or even ameliorate) cognitive functions during the MS course (see Sandroff et al. 2016 for a review). In fact, there is emerging evidence that CR may not be limited to premorbid factors (i.e., genetics, education and early-life behavior) that are not highly amenable to change but also could be impacted by training programs.

**Fig. 6:**
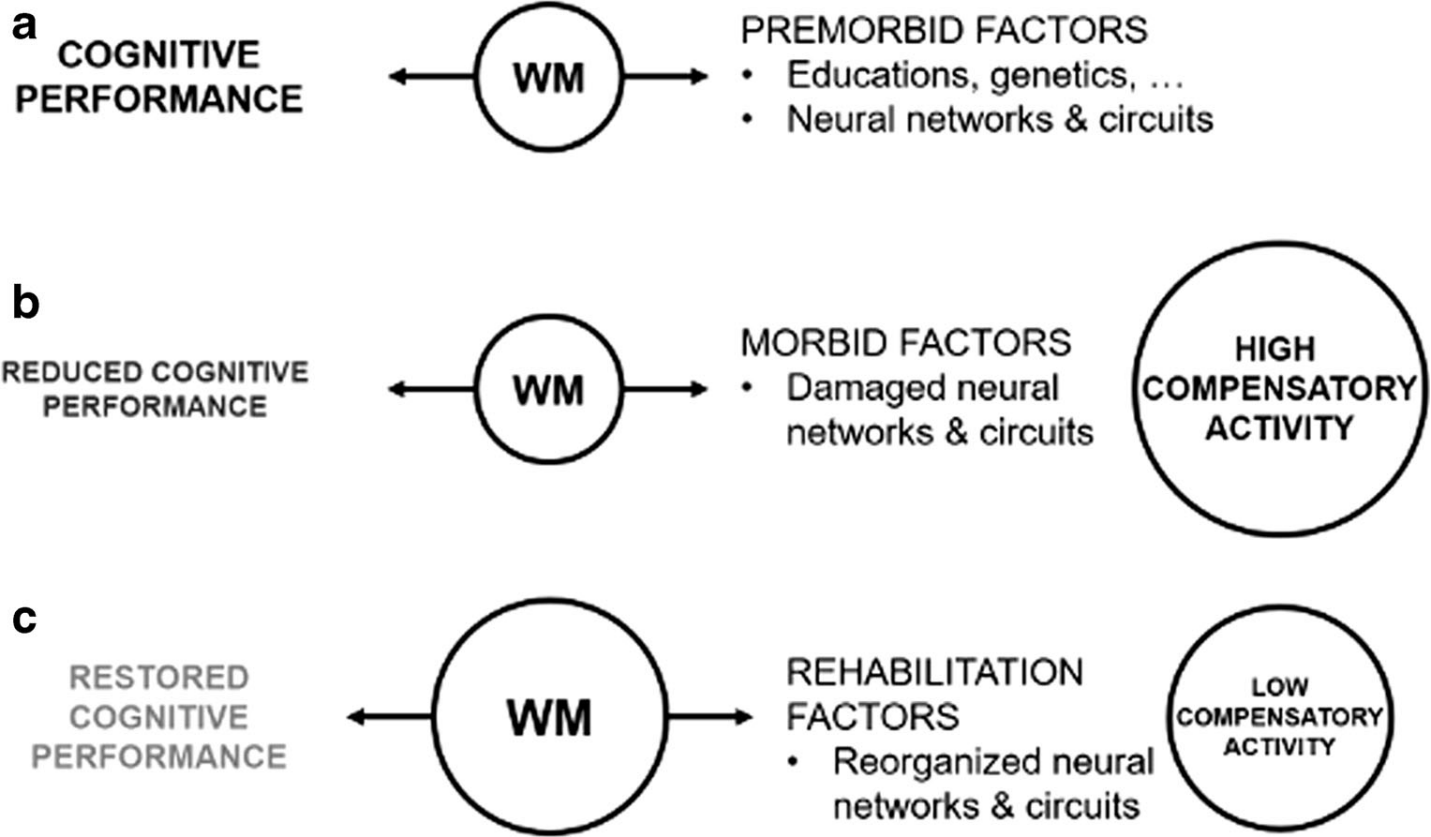
Theoretical framework of cognitive reserve functioning in MS. WM was shown to be a mechanism linking cognitive performance (in particular in a task of long-term memory) and individual factors (Sandry and Sumowski 2014) (**a**). In this and previous studies (Bonzano et al. 2009) we demonstrated that in PwMS brain damage leads to increased widespread neural activity, in spite of a reduced cognitive performance (**b**). However, an effective cognitive rehabilitation treatment can increase WM capacity, reduce compensatory activity and restore, at least in part, cognitive performance (**c**)

As previously suggested by Sandry and Sumowski, 2014, WM seems to act as a “memory buffer” mediating cognitive performance and individual aspects which can relate either to premorbid characteristics (e.g., genetics, education and neural functioning) or to disease-mediated factors, such as brain damage and dysfunctions (Fig. 6). As WM capacity increased following a specific, intensive and personalized training, compensatory areas that spontaneously activated in the attempt to maintain function reduced their activity. At the same time, overall cognitive performance was at least partly restored, as shown by the increased scores obtained by the patients at the neuropsychological battery after the intervention.

Limitations of the present study include the small sample size (which probably prevented us from obtaining significant results at a corrected threshold in the contrast analysis) and the lack of a control group of patients receiving a non-adaptive intervention. Also, the healthy group underwent only one assessment session. Moreover, while we did take into account in our analyses the differences in brain volume (a major determinant of cognitive performance), we did not correct for clinical variables nor treatments or white matter lesions, given the proof-of-concept nature of this work. Therefore, these results should be interpreted with caution and not generalized to the all population of PwMS.

A limitation regarding data interpretation is represented by the use of the PVSAT paradigm which is impacted by confounding factors such as anxiety and it is not a pure measure of a single cognitive function. Especially, PVSAT paradigm probes different constructs such as processing speed, attention and working memory, which are mediated by only partly overlapping neural networks (Olivers et al. 2011).

In conclusion, we suggest that an adaptive training based on WM (in this study administered by means of COGNI-TRAcK) can enhance the brain ability to cope with tissue damage to maintain or even partly restore cognitive functioning. In addition, this study showed that the right BA40 seems to be a “core” of functional reserve for cognitive performance in MS, since it was still active after the cognitive rehabilitation treatment (differently from other areas, such as the left BA 31) and its BOLD signal significantly correlated with cognitive performance. These findings open new perspectives on the role of the right BA40 in cognitive functioning in people with neurological disorders. In particular, further longitudinal studies should aim to monitor this area along the course of the disease or during longer rehabilitative treatments, in order to evaluate the efficacy of interventions also at a neural level.
